# Complete excision of a giant thyroid goiter in posterior mediastinum

**DOI:** 10.1186/1749-8090-8-207

**Published:** 2013-11-07

**Authors:** Xin Chen, Hongfei Xu, Yiming Ni, Ke Sun, Weidong Li

**Affiliations:** 1 Department of Thoracic and Cardiovascular Surgery, First Affiliated Hospital of Zhejiang University, School of Medicine, Qing Chun Road 79#, Hangzhou, China; 2 Department of Pathology, First Affiliated Hospital of Zhejiang University, School of Medicine, Hangzhou, China

**Keywords:** Intrathoracic goiter, Posterior mediastinum, Thoracotomy

## Abstract

Intrathoracic goiter is commonly located in the anterior mediastinum. Here we report a case of a 58-year-old Chinese male in whom we successfully removed the intrathoracic goiter and eased his dyspnea by a right posterolateral thoracotomy approach. Posterior mediastinal thyroid goiter with mediastinal compressive symptoms is an indication of surgery.

## Background

The intrathoracic thyroid adenoma or goiter is mostly located in the anterior mediastinum, about 10%-15% are in the posterior mediastinum [1]. It is derived from embryonic thyroid tissue and developing into isolated thyroid tumor within the mediastinum or descending into the retrosternal loose tissue space from neck, which may cause various compressive symptoms when it reaches a certain size. Most of the anterior mediastinal goiters can be removed by a transcerival approach, but posterior mediastinal goiters may require additional extracervical incisions [2].

## Case presentation

**Figure 1:**
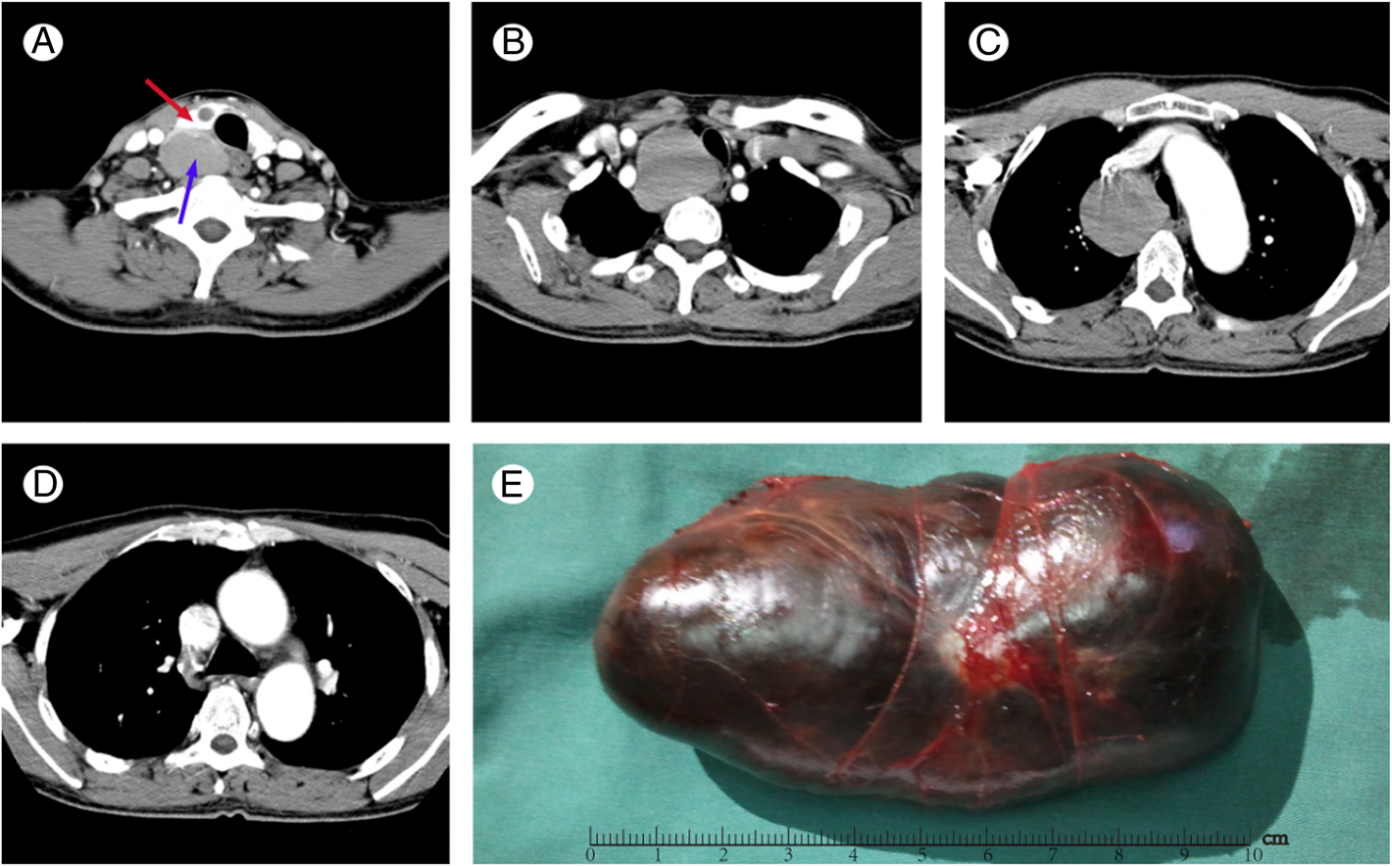
**CT scanning and complete excision of giant thyroid goiter in posterior mediastinum. (A)** Enhanced CT scanning reveals the right thyroid lobe (in red arrow) with a small cyst and a giant goiter (in blue arrow) in low density is on the back of the right lobe. **(B)** CT clavicle cross section reveals the giant goiter was located in the posterior mediastinum, compressing the trachea and esophagus. **(C)** CT of the chest reveals the goiter is well beyond the aortic arch and compressing the superior vena cava. **(D)** CT of the chest reveals the lower edge of the goiter reachs the carina of trachea. **(E)** The tumor is in a complete capsule with large tension, 10.0 × 9.0 × 9.0 cm in size.

A 58-year-old Chinese male was admitted to our hospital with a chief complaint of chest tightness and shortness of breath after activities for more than 4 months. Physical examinations show his heart rate of 96 beats/min, blood pressure of 130/80 mmHg, and no obvious mass in the neck. Hematological examinations show thyroid function was normal. Computed Tomography (CT) of the neck and chest showed a goiter of low density in the right thyroid, and a giant cystic nodule on the back of the right thyroid which grew into the right posterior mediastinum (Figure 1A). The tumor was located between the spine and the dorsal part of trachea and esophagus, its lower edge extended beyond the aortic arch and compressed the trachea to the left (Figure 1B-D). Other laboratory tests revealed no abnormalities. In March 2012, surgery was done by right posterolateral thoracotomy of the fourth intercostal space, a posterior mediastinal tumor (10.0 × 9.0 × 9.0 cm) fitting the location on CT was seen, and the right gland lobe was not excided. The mass was completely encapsulated with large tension (Figure 1E), and this cyst-solidary mass was hypervascular. Microscopy showed thyroid hyperplasia without malignancy. The final diagnosis was a secondary giant thyroid goiter in posterior mediastinum. In December 2012, the latest follow-up showd that patient now had no symptoms after activities or thyroid dysfunction.

### Discussion

Intrathoracic goiter refers to a goiter where most of its mass is found within the mediastinum. According to the originations of thyroid tissue, intrathoracic goiter can be divided into primary intrathoracic goiter and secondary intrathoracic goiter. The vast majority of intrathoracic goiters are secondary ones which arise from the lower part of one lobe or both lobes of cervical thyroid or isthmus and grow down through the thoracic inlet. Swallowing, gravity and thoracic negative pressure help the growing goiter direct into the chest cavity. Anatomically speaking, goiter in the chest cavity generally grows to the position of relatively low resistance. At first, the tumor will grow into the anterior superior mediastinum between trachea and sternum, forming the common retrosternal thyroid goiter. Because there are thymus (may atrophy), left and right brachiocephalic veins and superior vena cava in the front, aortic arch and its three branches (phrenic nerve and vagus nerve have smaller resistance) in the middle left of retrosternal space, tumor growth will be resisted there. Right posterior mediastinum has relatively low resistance than left posterior mediastinum, and it helps form right posterior mediastinal goiter. The primary intrathoracic goiter only accounts for 0.2 ~ 1% of all the intrathoracic goiters, it affects females more often (male: female = 1 : 3 or 1 : 4) [3]. Its causes are totally different from the ones of secondary intrathoracic goiter. During the embryonic developmental period of thyroid gland, part or all of the thyroid blastoma leaves primordium and is pulled into the thoracic cavity by the descendent heart and great vessels, then continues to develop in the thoracic cavity, forming the final primary intrathoracic goiter. Because of different originations, secondary posterior mediastinal goiter is often continued with the cervical thyroid gland, with blood supply from inferior thyroid artery and its branches while primary posterior mediastinal goiter maintains little or no connection with the cervical thyroid gland, and has a blood supply derived from intrathoracic arteries [4].

Patient generally has no symptoms when the goiter is small, many cases are only found in occasional chest radiographic examination or autopsy. As the goiter increases in size, a variety of clinical symptoms may appear due to compression of surrounding organs and tissues (i.e. trachea, esophagus, lungs, or even superior vena cava). Most investigators agree that respiratory symptoms are caused by compression of the airway [5]. Thyroid function test has a low susceptibility in predicting goiter, for most patients are normal and only 10 ~ 15% show hypothyroxinemia.

Radiographic image is the most effective and necessary diagnostic method for intrathoracic goiter. CT scan is the most common one for preoperative evaluation. On CT films, intrathoracic goiter usually manifests as a clear boundary mass, its density varies due to the amount of iodine contained: when the amount of iodine in the mass is low, its density is close to the soft tissue of chest wall, and when the amount of iodine is high, its density could be greatly higher than soft tissue. In addition, its density can be uneven due to colloid cysts and calcified plaque. Radionuclide scan is also one of the common diagnostic methods, but it is not so effective when compared with its usage in thyroid goiter of other regions because the intrathoracic goiter does not always uptake iodine.

The differential diagnosis of intrathoracic goiter are of great variety, it should be differentiated from lymphadenopathy, branchial cleft cyst, arterial aneurysm, neurogenic tumour, pheochromocytoma, spinal cord injury, hiatus hernia, etc.

When the trachea, esophagus or vena cava is compressed, surgical resection of intrathoracic goiter must be done. Preventive operation is also feasible for asymptomatic patients in order to avoid future compression. Secondary intrathoracic goiter is always taken out through inferior cervical collar incision, but posterior mediastinal goiters which extended beyond the aortic arch may require additional extracervical incisions [6]. A variety of operation modes exist, including sternotomy, clavicular resection, anterior posterolateral thoracotomy and Video Assisted Thoracoscopic Surgery (VATS). A specific mode depends on the location, size of the mass and its relationship with surrounding important organs. Attention should be payed to some special points during anesthesia. Similar to the anesthesia of mediastinal giant tumors, intravenous anesthesia combined with tracheal intubation is used. But because the tumor is located in the posterior mediastinum, compressing adjacent organs, especially the carina, plus various factors such as mental stress, thick sputum or postural changes, extreme hypoxia or heart arrest may occur at any time, resulting in failure of anesthesia induction and tracheal intubation, in which cardiopulmonary bypass (CPB) may be needed. When patient needs to be changed to lateral position after anesthesia induction, compression of heart or great vessels by the tumor should be watched out. Chest must be opened as soon as possible to decompress the heart when cardiac output decreases and blood pressure drops sharply. Common surgical complications include postoperative airway collapse, respiratory tract infection and bleeding [7].

## Conclusions

Posterior mediastinal goiter with mediastinal compressive symptoms is an indication of surgery. Lateral thoracotomy is an alternative approach for intrathoracic goiter extending into the posterior mediastinum.

## Consent

Written informed consent was obtained from the patient for publication of this Case report and any accompanying images. A copy of the written consent is available for review by the Editor-in-Chief of this journal.

## Abbreviations

CT: Computed tomography; VATS: Video assisted thoracoscopic surgery; CPB: Cardiopulmonary bypass.

## Competing interests

The authors declare that they have no competing interests.

## Authors’ contribution

XC wrote the article, HFX and YMN collected the clinical information, KS selected the images and carryed out the diagnosis, WDL drafted the final manuscript. All authors read and approved the final manuscript to be published.
